# Immunomodulatory Effect of Microglia-Released Cytokines in Gliomas

**DOI:** 10.3390/brainsci11040466

**Published:** 2021-04-07

**Authors:** Marika Lanza, Giovanna Casili, Michela Campolo, Irene Paterniti, Cristina Colarossi, Marzia Mare, Raffella Giuffrida, Maria Caffo, Emanuela Esposito, Salvatore Cuzzocrea

**Affiliations:** 1 Department of Chemical, Biological, Pharmaceutical and Environmental Sciences, University of Messina, Viale Ferdinando Stagno D’Alcontres, 98166 Messina, Italy; mlanza@unime.it (M.L.); gcasili@unime.it (G.C.); campolom@unime.it (M.C.); ipaterniti@unime.it (I.P.); salvator@unime.it (S.C.); 2 Mediterranean Institute of Oncology, Via Penninazzo 7, 95029 Viagrande, Italy; cristina.colarossi@grupposamed.com (C.C.); marzia.mare@grupposamed.com (M.M.); 3 IOM Ricerca Srl, Via Penninazzo 11, 95029 Viagrande, Italy; raffaella.giuffrida@grupposamed.com; 4 Department of Biomedical and Dental Sciences and Morpho-Functional Imaging, Unit of Neurosurgery, University of Messina, 98122 Messina, Italy; mcaffo@unime.it

**Keywords:** microglia, immunotherapy, brain tumors

## Abstract

Microglia, a type of differentiated tissue macrophage, are considered to be the most plastic cell population of the central nervous system (CNS). Microglia substantially contribute to the growth and invasion of tumor mass in brain tumors including glioblastoma (GB). In response to pathological conditions, resting microglia undergo a stereotypic activation process and become capable of phagocytosis, antigen presentation, and lymphocyte activation. Considering their immune effector function, it is not surprising to see microglia accumulation in almost every CNS disease process, including malignant brain tumors. Large numbers of glioma associated microglia and macrophages (GAMs) can accumulate within the tumor where they appear to have an important role in prognosis. GAMs constitute the largest portion of tumor infiltrating cells, contributing up to 30% of the entire glioma mass and upon interaction with neoplastic cells. GAMs acquire a unique phenotype of activation, including both M1 and M2 specific markers. It has been demonstrated that microglia possess a dual role: on one hand, microglia may represent a CNS anti-tumor response, which is inactivated by local secretion of immunosuppressive factors by glioma cells. On the other hand, taking into account that microglia are capable of secreting a variety of immunomodulatory cytokines, it is possible that they are attracted by gliomas to promote tumor growth. A better understanding of microglia-glioma interaction will be helpful in designing novel immune-based therapies against these fatal tumors. Concluding, as microglia significantly may contribute to glioma biology, favoring tumor growth and invasiveness, these cells represent a valuable alternative/additional target for the development of more effective treatments for gliomas.

## 1. Introduction

There are over 100 types of cancer that can affect the central nervous system (CNS) [1]. Glioblastoma is a particularly aggressive type of cancer affecting the glial cells, in particular astrocytes, which have a supporting role in CNS [2]. Furthermore, it represents about 45.2% of all malignant CNS tumors [3,4] with an incidence of 5–6 cases per 100,000 people [5].

Glioblastoma is usually described in two different clinical forms, primary and secondary; primary glioblastoma is the most common form (about 95%) and arises typically de novo, within 3–6 months, in older patients, while secondary glioblastoma arises from prior low-grade astrocytomas (over 10–15 years) in younger patients [6]. Glioma is the most common form of CNS neoplasm that originates from glial cells. Malignant gliomas are the most common and deadly brain cancers of the CNS. In the 2007 World Health Organization (WHO) classification, the main glial tumor groups included: astrocytic tumors, oligodendroglial tumors, oligoastrocytic tumors, ependymal tumors, and neuronal and mixed neuronal-glial tumors (such as gangliogliomas). These groups included more circumscribed grade I tumors such as pilocytic astrocytomas, pleomorphic xanthoastrocytomas, and subependymal giant cell astrocytomas, as well as the more common infiltrating gliomas, including grade II oligodendrogliomas and astrocytomas, and grade III anaplastic oligodendrogliomas, anaplastic astrocytomas, anaplastic oligoastrocytomas, anaplastic ependymomas, and grade IV glioblastomas (GBM) [7]. Malignant primary brain tumors represent a leading cause of cancer mortality in children and young adults, with few therapeutic options. In adults, glioblastomas, the most common primary brain tumors, remain uniformly lethal, with a median survival of <21 months, despite surgical resection, targeted radiation therapy, high-dose chemotherapy, and novel approaches such as tumor-treating fields (TTFields) [8,9,10,11,12,13,14,15]. In this regard, Laser interstitial thermal therapy (LITT) is an emerging minimally invasive procedure increasingly utilized for treatment of deep and recurrent glioblastoma (GB), in that provides an effective treatment with low morbidity for selected patients harboring recurrent GB. LITT could be included in the armamentarium of neurosurgical oncologist for treatment of recurrent GB [16].

Glioblastoma (GB), the most aggressive malignant glioma, contains high levels of microglia infiltrates [17]. About 30% of the tumor mass is composed of glioma-associated microglia and macrophages (GAMs), which suggests that innate immune cells play an important role in the pathology of GB.

The immune system in the brain follows different principles from the immune system elsewhere, whereby access to the tumors is limited by the blood–brain barrier (BBB), and the host is subjected to substantial endogenous and treatment-induced immune suppression. Despite the other tumor types, CNS tumors display low numbers of tumor-infiltrating lymphocytes (TILs) and other immune effector cell types [18]. The reduced quantity and limited activity of T cells in CNS tumors is largely owing to the unique immune environment of the brain [19]. GB is composed of different types of cells, including glioblastoma stem cells (GSCs) that are responsible for tumor malignancy and expansion [20]. Other types of cells that are also present in the tumor mass include astrocytes, oligodendrocytes, endothelial cells, neurons, microglia, and tumor-infiltrating macrophages, which are the most common cells within GB [19,21,22]. Microglia are mostly present in the gray matter, particularly in the hippocampus, olfactory telencephalon, basal ganglia, and substantia nigra. The distribution of these cells in adult mouse brains fluctuates from 5% in the cortex to 12% in the substantia nigra [23,24]. Particularly, recent studies on human brains observed that microglial self-renewal was maintained by coupled proliferation and apoptosis with about 0.5–2.5% of proliferation rate, that varied across regions in the young adult mouse brain, with microglia in the dentate gyrus having the highest [25,26,27].

In the tumor, microglia cells can polarize into two different phenotypes, the classic M1 and the alternative M2 phenotype [28]. The M1 phenotype is functionally characterized by its ability to eliminate microorganisms or tumor cells, and to produce proinflammatory cytokines, such as interleukin (IL)-1β, IL-6, Tumor necrosis factor (TNF)-α, among others [29], while M2 phenotype is associated with prolonged neural survival, restriction of brain damage, and prevention of destructive immune responses [30]. Cytokines constitute a significant portion of the immuno- and neuromodulatory messengers released by activated microglia [31]. In principle, microglial activation aims at CNS protection; however, failed microglial engagement due to excessive or sustained activation that could significantly contribute to acute and chronic neuropathologies. Thereby, dysregulation of microglial cytokine production could promote harmful actions of the defense mechanisms, resulting in neurotoxicity, as well as disturb neural cell functions as they are sensitive to cytokine signaling. The role of microglial cells is to detect and eliminate any insults that may affect the normal brain function. Specifically, microglia express high levels of P2RY12, which serve as chemotactic receptor at sites of CNS injury. Microglia can aggregate and form a physical barrier, via E-cadherin upregulation, that for example will engulf the damaged vessel wall and temporarily assume blood–brain barrier functions. In homeostatic conditions, microglia remain resting or quiescent, but in response to insults, including tumor cell invasion, they become activated. These cells can adopt many different states of polarization during cancer progression that fluctuates across a pro- to anti-inflammatory spectrum. The immune cells with a pro-inflammatory phenotype have been described as exerting an anti-tumorigenic effect, whereas those with an anti-inflammatory profile showed tumor-supporting activity [32,33].

## 2. The Microenvironment of Glioblastoma

In the last decade, many studies have been conducted to understand the role of genetic mutations and microenvironment in glioblastoma tumorigenesis [34,35,36,37]. The TME is composed of both the noncancerous cells and biomolecules inside the tumor as well as the extracellular matrix (ECM). Noncancerous cells constituting the TME include normal and reactive astrocytes, GBM stem cells (GSCs), fibroblasts, vascular pericytes, immune cells, microglia/macrophages, and endothelial cells (ECs). Biomolecules produced by noncancerous cells include cytokines, chemokines, hormones, and nitric oxide (NO). One notable characteristic of GBM is hypoxia occurring in the whole tumor with variable intensity within the TME, it is considered to be an efficient hallmark of GBM [38]. The number of glioma-associated microglia/macrophages (GAMs) is almost equal to the number of tumor cells. Compelling evidence has proven that GAMs favor tumor progression, since GAMs together with other myeloid cells are strictly related to the immunological features of gliomas [39]. For instance, GAMs secrete TGF-β that promotes GBM cells release ofversican, MMP2, and MMP9, the matrix metallo proteases critical for the degradation of ECM components such as collagen and elastin to enhance the invasiveness of GBM [40]. Astrocytes, one main component of the GBM microenvironment, display a reactive phenotype when they contact tumor cells. A large amount of growth factors, chemokines, cytokines, and other soluble substances secreted by reactive astrocytes serve as essential inter-mediators orchestrating continued astrocyte activation and signaling transfer between stroma and epithelial GBM cells. Therefore, reactive astrocytes are an important source of secretions in the TME of GBM, augmenting GBM malignancy by causing aberrant cell proliferation and triggering a malignant transformation in the tumor microenvironment [41]. Astrocytes represent one main component of the Neuroglia [42]. Astrocytes closely interact with surrounding structures in the nervous system and contribute to the regulation its functions, wrapping up neuronal somas, axons, dendrites, and synapses [42]. Astrocytes are divided into two main subtypes: “protoplasmic”, located in the substance gray brain, and “fibrosis”, located mainly in the white matter, so-called for the presence of the acid fibrillar protein of the glia (GFAP), involved in the structure and function of the cell cytoskeleton [43]. Generally, the astrocytes fibrosis are characterized by longer and thinner filaments, instead the astrocytes protoplasmatic have branched and developed short filaments [44]. Recent studies revealed that astrocytes control the cerebral blood flow, promote the absorption of glucose, the only source of energy for neurons, and guarantee the optimal functioning of neuronal cells [44]. In addition, they play a key role for maintaining CNS homeostasis, being involved in the metabolic cycle of GABA and glutamate [44]. A common feature in many CNS diseases is astrogliosis, or reactive gliosis. In this context, astrocytes undergo conformational and functional changes, activated by pro-inflammatory molecules released from microglia to counteract cerebral damages [44]. Reactive gliosis is an important process because it gives rise to multiple neurotrophic factors, capable of ensuring the survival of neuronal cells, characterized also by a strong astrocytic proliferation [44]. Astrogliosis protects the CNS from further damage cerebral, however increasing evidence indicates that dysfunctions of reactive astrogliosis process can contribute to, or to be primary causes of, CNS diseases and brain tumors through loss of their normal functions [44].

## 3. Immune Component of CNS: Microglia

**Figure 1:**
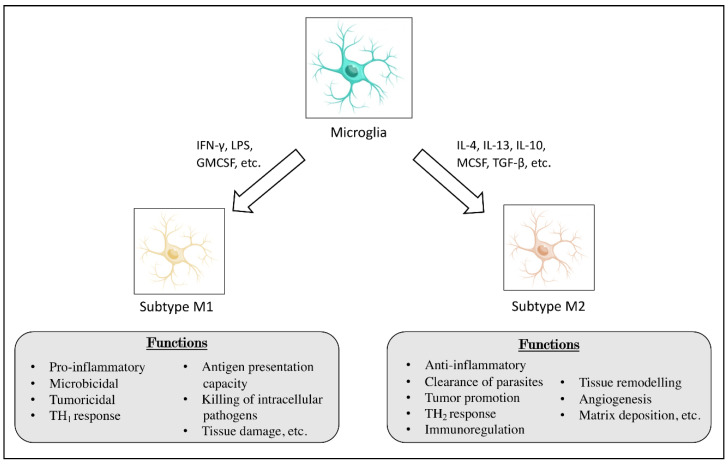
Differentiation of microglia. The figure illustrates the differentiation of microglia into M1 and M2 subtypes, with the related functions for each.

The brain parenchyma is populated by microglia which represents the innate immune component of the CNS [45,46]. Microglia comprise ~10–15% of all glial cells present in CNS and are often referred as the tissue-resident macrophages of the CNS [47]. Microglial cells play an important role in maintaining normal brain function, able to prevent pathogenic invasion, neuroinflammation, or brain damage [48]. Microglial cells are able to communicate directly with neurons, astrocytes, and blood vessels [45]. Moreover, it has been shown that microglia play an unexpected roles in normal brain development and adult physiology [48], as well as the involvement in several physiological functions, such as phagocytic activity and cytokines production [45]. As shown in several studies [49,50,51,52], microglial cells can be differentiated into classical (M1) or alternative (M2) phenotype by microenvironment stimuli. (Figure 1) M1 cells are activated by type I cytokines such as interferon-γ (IFN-γ), TNF-α, as well as by lipopolysaccharide (LPS), and lipoproteins. Microglial morphology varies throughout the brain depending upon tissue type, region, age, sex, and presence or absence of immune challenge or injury [48]. Under physiological conditions, microglia are in a resting state characterized by ramified morphology; however, upon exposure to infectious and traumatic stimuli, microglia rapidly change their morphology to “amoeboid” activated phenotype, producing various substances such as reactive oxygen species (ROS), and nitric oxide, pro-inflammatory cytokines, and chemokines, which contribute to the clearance of pathogenic infections [46,53]. In this context, microglial cells are activated by any type of pathologic event or change in brain homeostasis, inducing genetic programs designed to overcome and repair CNS insults [48]; however, this activation process is highly diverse and depends on the context and type of the stress or pathology [45]. Transformations of microglial morphology, phenotype, and function are observed during almost all neuropathological conditions such as degenerative diseases, infection, tumors and brain injury [46]. Microglial cells, following recognizing pathogen- and danger-associated molecular patterns on pathogens, are able to regulate programmed cell death, strongly influencing the pathologic outcome or response to a stressor due to the release of several substances, including growth factors, cytokines and chemokines [45,53] which are important for trafficking immune cells in the central nervous system [53,54]. The activation of immune system in the primary tumor might influence the multifactor process of cancer progression [55]. Particularly, microglial activation results in the production of pro-inflammatory cytokines such as IL-1, IL-6, and TNF-α. Although the release of pro-inflammatory cytokines is typically intended to prevent further damage to CNS tissue, they may also be toxic to neurons and other glial cells, thus contributing to the development and progression of several diseases as brain tumors.

## 4. Role of Microglia in Brain Tumors

**Figure 2:**
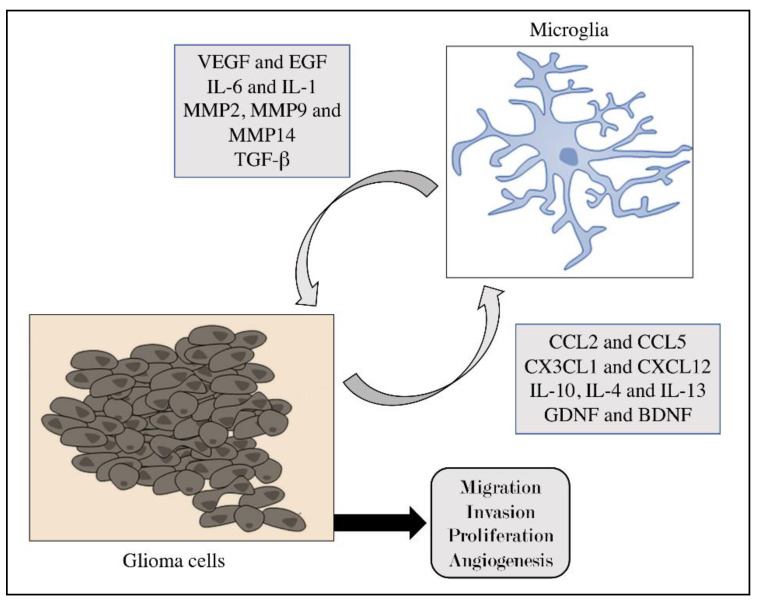
Cross-talk between glioma cells and microglia.

A prolonged and chronic microglial activation may induce a marked inflammation that could contribute to progression of neurodegenerative and neoplastic diseases [56,57,58,59]. M1 microglial subtype performs an anti-tumor immune function by producing pro-inflammatory cytokines, ROS, and express signal transducer and activator of transcription 1 (STAT1). In particular, STAT1 plays a key role in cell growth and apoptosis, as it acts as a tumor suppressor by altering the function of protein, DNA, or RNA, or by inducing lipid peroxidation leading to tumor growth inhibition [60,61]. On the contrary, M2 cells are activated by type II cytokines such as IL-4, IL-10, IL-13, and transforming growth factor (TGF)-β, performing a pro-tumor immune response by producing factors as STAT3 [62], which contribute to tumor proliferation [60,63]. (Figure 2) Specifically, STAT3 is considered a crosstalk between cancer and immune cells. It is constitutively activated both in tumor cells and in immune cells of the tumor microenvironment. STAT3 is able to inhibit the expression of mediators required for immune activation against tumor cells, promoting tumor progression [64]. Microglial cells become hyper-activated through two mechanisms in brain tumor microenvironment; first of all, microglial cells become active, produce cytokines, growth factors and matrix-metalloproteases (MMPs) in response to initial tumor cell stimuli which promote tumor growth and invasion [57]. Second, tumor cells release chemoattractant, and chemokine factors that recruit and induce another wave of microglial activation, resulting in a perpetuating cycle of microglia activation in the brain tumor, increasing its growth [56]. Specifically, in response to brain tumor microenvironment, resident microglia or macrophages infiltrating from the circulation become polarized towards a pro-inflammatory (M1) phenotype upon exposure to pro-inflammatory cytokines IFN-γ, TNF-α, and cellular debris. Then, these cells present antigen through MHC class II protein, produce pro-inflammatory cytokines as interleukin IL-1β, IL-12, and express high levels of inducible NO (iNOS) essential for NO production [65]; these actions by M1 phenotype are essential for activating T cells and the consequent adaptive immune response against brain tumor cells [65]. Conversely, M2 polarization prevents the production of cytokines required to support tumor-specific CD8+ T cells, and CD4+ T helper 1 (Th1), promoting tumor proliferation. Furthermore, M2 polarization is characterized by the expression of surface CD163 and CD204, expression of intracellular STAT-3 and the production of arginase, IL-10, and transforming growth factor beta 1 (TGF-β1), supporting tumor invasion and angiogenesis [66]. Although the identity of tumor-derived cytokines/chemokines that modulate the recruitment of microglia remains unclear, several common chemokines and receptors have been found to be up-regulated in brain tumors, including monocyte chemoattractant protein-1 (MCP-1), granulocyte/macrophage-colony stimulating factor (GM-CSF), fractalkine (CX3CL1), and CCL [67,68]. In particular, MCP-1/CCL2 is believed to be a major contributor in microglia recruitment to gliomas and others brain tumor metastases [57,68]. Many studies have focused on the controversial role of microglia in brain tumors, showing a dual role for tumor progression [60,69,70]. The tumor microenvironment is complex and includes neoplastic cells as well as varieties of host and infiltrating immune cells as microglial cells [69]. As shown in a study on glioma conducted by Bettinger et al., microglial cells are part of the glioma microenvironment, playing a critical part in initiating and maintaining tumor growth and progression [69,71]. Moreover, many studies showed that microglia-derived enzymes, cytokines, growth factors can increase brain tumor proliferation through numerous processes such as immunosuppression, inflammation, and angiogenesis [72,73]. A lack of microglia/macrophages has been observed to significantly affect metastatic spread of tumors, suggesting that these two types of cells play an essential role in the brain tumor invasion and metastasis [60]. In particular, angiogenesis is considered a key event in tumor growth and progression; in this context, microglial cells can exert an important influence on blood vessel formation and function associated to production of angiogenic factors as vascular endothelial growth factor (VEGF) which stimulates angiogenesis promoting tumor growth [74]. Recently, Blank et al. showed that microglia/macrophages of the GBM-patients were highly associated with tumor blood vessels, accompanied by remodeling of the vascular structure, emphasizing that tumor-infiltrating myeloid cells might represent a crucial part for limited efficacy of anti-angiogenic therapy [75]. Moreover, also inflammation represents a key event in brain tumor progression leading to production of chemokines as CXCL12, and CXCL8, reactive oxygen species (ROS) and nitrogen (RSN), extremely reactive molecules which promote the development of tumors by damaging DNA, proteins and lipids [76]. Belonging to the subfamily of the CXC chemokines, CXCL10 is shown to be proinflammatory and proliferative and is associated with advanced human cancer [77]. Although the identity of tumor-derived cytokines/chemokines that modulate the recruitment of microglia remains unclear, several common chemokines and receptors have been found to be up-regulated in brain tumors, including monocyte chemoattractant protein-1 (MCP-1) [60]. Thus, on the basis of these scientific evidences, a possible inhibition of microglial activation could symbolize a suitable target to restrain inflammation or to limit brain tumor progression, representing a potential anti-neoplastic-targeted therapy to block the growth of brain tumor [56].

The cancer stem cell (CSC) hypothesis suggests that neoplastic clones are maintained exclusively by a rare fraction of cells with stem cell properties. One of the major limitations to our current understanding of cancer stem cells is the lack of definitive markers that can be used distinguish cancer stem cells from other cells in the tumor. As shown in a study by Singh SK et al. [78], CD133 (also known as prominin) can be used to identify and isolate cancer stem cells and these cells have a unique capacity to initiate tumor growth. However, low-grade gliomas, and a significant proportion of glioblastomas do not contain CD133+ cells [79,80], raising the issue of what cells give rise to these tumors. Possible candidates are glial progenitors and progenitor-like glioma cells, which are found in abundance in both low- and high-grade gliomas [81].

## 5. The Dual Role of Microglia in Tumors

The recently discovered pathway that controls the baseline motility of microglia processes is an interesting therapeutic avenue to pursue for the treatment of GB. When microglia are challenged, like in the case of tumor formation, their immunological response can be strikingly suppressed or maladapted [82]. It is now well recognized that microglia have functional plasticity and dual phenotypes, proinflammatory M1, and anti-inflammatory M2 phenotypes. The M2 state is further subdivided into M2a, M2b, and M2c, with expression of the anti-inflammatory cytokines as well as arginase-1 (Arg1), TGF-β, CD206, and Chitinase-3-like-3 (Ym1 in rodents). These three states have some biochemical overlap with distinct activation mechanisms. It is reported that STAT6 and STAT3 pathways are involved in the activation of M2a and M2c, while the signaling pathway that involves M2b activation is largely unknown [28,83,84]. The evidence of microglia polarization in neuroinflammation-related diseases models led to the idea that, compared with merely suppressing microglia activation, the inhibition of M1 microglia polarization along with promoting M2 microglia polarization may be a viable treatment strategy for the treatment of inflammation-related diseases [85,86,87]. Moreover, activated M2 microglial cells have been shown to promote colonization of breast cancer cells in the brain [88]. In contrast, activated M1 microglial cells induced apoptosis of metastatic lung cancer cells in vitro [89]. Both M1 and M2 microglia are detected within brain tumor mass; even if M1 microglia is able to suppress tumor growth and cause tumor cell death, the immunological functions of M1 microglia in the brain tumor including cytotoxicity, phagocytosis, and antigen presentation are impaired [90]. Therefore, these microglia/macrophages can be polarized into becoming tumor-supportive and immunosuppressive cells by certain tumor-derived soluble factors, thereby promoting tumor maintenance and progression.

## 6. Current Primary Therapies for Gliomas

The three main treatments for high grade gliomas have remained consistent for the past 3 decades: maximal surgical resection, radiation therapy, and chemotherapy. Initial therapy consists of maximal safe surgical resection. Multiple retrospective analysis have supported the importance of gross total resection of the contrast enhancing tumor, with longer overall survival seen in patients with greater extent of resection [91,92,93]. Therefore, pre-operative imaging is a mainstay for safe and effective surgical resection. Eloquent cortex is now mapped with several different imaging techniques including functional MRI (fMRI), magnetoencephalography (MEG), navigated transcranial magnetic stimulation (nTMS), and diffusion tensor imaging fiber tracking (DTI-FT). Novel intraoperative techniques to improve extent of resection include the use of fluorescent marker 5-amuionlevulinic acid with operation under blue light [94,95]. Tumor cells preferentially take up this florescent dye, enhancing the visualization of tumor using filtered light to aid maximal resection. It is administered orally, 2 to 3 h prior to surgery, and reaches maximal florescence within the tumor cells at 6 h [96]. Florescence is correlated with histologic tumor grade, and structures such as blood vessels, normal brain tissue, olfactory trace, and the dura do not fluoresce [96]. Limitations of this technique include uptake by the choroid plexus, ventricular ependymal, and pia mater, making ventricular tumor margins more complicated as well as photo-bleaching in white light [96]. Over the years, radiotherapy (RT) has increasingly assumed a fundamental role in the treatment of primary brain tumors as well as metastases [97]. Indeed, thanks to advances in imaging and radiotherapy techniques, it has been possible to make a more precise localization of the tumor, thus allowing a reduction in the volume of irradiated healthy brain tissue [97]. All this led to a reduction in long-term toxicity due to radiotherapy, but with the same results in terms of efficacy. Fractionated localized radiation is a mainstay of therapy for all GBMs. The standard dose of radiation, 60 gy, delivered in 30 to 33 fractions of 1.8–2 gy is delivered to the involved field as well as 1–3 cm of margin to treat infiltrating tumor. In older patient populations with worse performance status, hypo fractionated regimens (ex 40 gy in 15 fractions) can be utilized. Defining tumor margins has remained a challenge for both surgery and radiation therapy. The role of new imaging techniques has begun to better define tumors; however, its role in surgical planning and radiation therapy varies. Imaging is now being used to characterize brain tumor biology, guide therapy, assess therapeutic response, detect early treatment failure, predict clinical outcomes, and can be used to distinguish between tumor progression and treatment effects. Currently, the standard of care for treatment of high-grade gliomas includes the use of temozolomide an oral cytotoxic DNA-alkylating chemotherapy. Use of temozolomide with concomitant radiation therapy followed by adjuvant temozolomide for 6 months has been shown in a large phase 3 clinical trial to improve median overall survival compared to radiation alone (14.6 months compared to 12.1 months), with a twofold increase in 2-year survival from 10.4 to 26.1%. The standard of care for low grade glioma remains somewhat in dispute. There remain many limitations to the current chemotherapeutics in use for glioma. Systemically delivered medications typically do not reach high concentrations within the CNS and at the site of the tumor. Furthermore, they lead to significant systemic side effects such as myelosuppression. Therefore, there is active research into novel drug delivery systems to improve the distribution of the agents directly to the brain tumor. One such area of research is the use of nanoparticles to aide in drug delivery. Nanoparticles can also be engineered to target tumor cells, and therefore minimize damage to healthy cells. The benefit of this technology is that it can efficiently deliver therapeutics in both a targeted and concentrated manner to tumor cells.

## 7. Immunotherapy in Brain Tumors by Cytokines Modulation

A hallmark of cancer is immune suppression, and brain tumors have been characterized as immunosuppressive [38]. Multiple lines of research into immunotherapy strategies are underway, including include cellular, vaccination, and immunomodulatory therapies targeting immune checkpoints. Immunotherapy, with the use of monoclonal antibody, in the last decades represent an innovative approach in the context of tumors, by modulation of biological response such as interferons and cytokines, vaccines, and more [98]. Immunotherapy can induce, amplify, or suppress an immune response from the body; in this regard, based on the pathology to be treated, we can distinguish two types of immunotherapy, a suppressive or activating type. This last type can induce or amplify the immune response [99]. Among the various forms of cancer, brain tumors can certainly be classified as the most lethal and widespread [100]. Currently, conventional therapy includes chemotherapy, radiotherapy, and surgical practice [100]. Therefore, considering the high mortality and the absence of an effective and resolutive therapy, the discovery of new therapeutic strategies for brain tumors would certainly represent an important goal for research. Reardon et al. [101] described how single-agent PD-1 blockade with nivolumab improves survival in patients with recurrent GB compared with bevacizumab, in a clinical study including 369 patients and having the overall survival (OS) as a primary endpoint. In another clinical study, Wang et al. [102], reported antigen-specific T cell responses in patients with GB immunized with dendritic cell vaccines pulsed with individualized tumor-associated antigens (TAA). Moreover, the results obtained by this study clearly demonstrated how TAA immunization-induced-specific CD4+ and CD8+ T cell responses, increase overall survival [102]. Sagnella et al. [103] studied an immunotherapic strategy by using proved the tumor-targeting role of EnGeneIC Dream Vector (EDV) nano cells in patients with GB. Many researches have suggested that immunotherapy is an attractive complement to conventional therapeutic modalities. Glioma research has already seen the clinical impact of high-dimensional of the tumor tissue and its immune components on classical biomarkers; for example, the cytologically diagnosable 10q23 deletion as a biomarker of tumor etiology and prognosis preceded elucidation of the tumor suppressor functions of PTEN and their cross-talk with glioma-specific growth pathways. Furthermore, EGFR expression, genomic amplification, and truncation were each associated with differential prognosis and therapeutic response in tumor cell growth and angiogenesis. Still, hypermethylation of O6-methylguanine DNA methyltransferase (MGMT) predicted increased sensitivity to temozolomide and radiation and isocitrate dehydrogenase-1 mutation (IDH1-R132H), which was a known biomarker in GBM [104]. There was early skepticism regarding whether elements of the peripheral, adaptive immune system could gain sufficient access to tumors in the CNS to have a significant impact on tumor growth. However, recent studies have clearly shown that immunization in the periphery can promote a therapeutically meaningful attack against well-known CNS tumors [105]. The immune response can be augmented by genetic modification of tumor cells to secrete cytokines, including IL-2, GM-CSF, and IFN-γ. Furthermore, the modification of MHC, to induce an express allogeneic answer, could be an interesting therapeutical method. In some cases, objective evidence of tumor regression has been observed in patients receiving immunizations only with tumor cell immunogens, suggesting the potential effectiveness of this type of immunotherapy for malignant neoplasms [106]. Recent advances have led to the identification of numerous cytokines released by microglia that modulate immune response involved in anti-tumor immunity. Several of these cytokines have been produced by recombinant DNA methodology and evaluated for their anti-tumor effects. In clinical trials, the administration of cytokines and related immunomodulators has resulted in independent tumor responses in some patients with various types of neoplasms [107]. Although recombinant cytokine proteins have been employed as biological drugs for cancer, systemic delivery of pharmacological doses of proteins often results in severe side effects and toxicities [108]. Therapeutic proteins tend to have very short half-lives and are complex to manufacture and deliver. Therefore, many investigators are evaluating the delivery of cytokine genes [109]. Physiologically, most cytokines are rather potent proteins acting at small quantities. This approach is utilized as a means of inducing systemic responses that can target residual primary lesions, as well as metastatic lesions. Therapeutic effects of cytokine gene transfer in preclinical cancer models in general, capable of allowing or inducing rejection of well-established tumors, have been demonstrated with IL-2 [110,111], GM-CSF [112], and type I IFNs such as IFN-α [113,114,115], IL-4, IL-12 [116], IL-18 [117], and IL-23 [118]. With regard to the application of cytokine gene therapy strategies to brain tumors, cytokine genes have been delivered:

(1) to peripheral vaccination sites in the form of cytokine gene transfected tumor cells as a means to activate systemic immune responses against brain tumors;
(2) directly to the brain tumor site to inhibit tumor growth and/or to enhance local antitumor immune responses and anti-angiogenic effects against the tumor.

Therefore, it is established that a better understanding of how microglia work is essential for the development of immune-based treatment strategies against malignant brain tumors. As highlighted by Acker and colleagues [119], CXCR2/CXCL2 signaling pathway represents a promising therapeutic approach in GL261 glioma cells by blocking microglia/macrophages activation. The effect of glioma cells and microglia was also analyzed by Badie et al. [120], the deepening of hepatocyte growth factor/scatter factor (HGF/SF) made it clear how these factors plays a role in glioma motility and mitogenesis, may also act as a chemokine for microglia and may be responsible for the microglia infiltration in malignant gliomas.

Other studies described how glioblastoma-associated macrophages and microglia are the predominant factors in the tumor microenvironment by elucidating the role of MerTK in this context [121], clarifying the involvement of epidermal growth factor receptor (EGFR) and colony stimulating factor 1 receptor (CSF-1R) signaling [122], and the activity of microglia by AAV2-mediated IL-12 through TRAIL [123] as well as the role of TNF-α [124]. (Table 1).

Future investigations should be directed towards determining the most suitable cytokine for each site (i.e., peripheral vaccines or brain tumor site) and timing (i.e., priming non-antigen experienced precursor cells or boosting vaccine-activated effectors) within the immunological milieu that can lead to tumor regression.

## 8. Conclusions

Despite countless scientific advances, current glioblastoma treatments have not improved the survival rates of patients. Targeted therapies have been shown to have limited efficacy as the pathophysiological mechanisms of brain tumors are still not fully understood. The combination of conventional therapies consisting of surgery, radiotherapy, and chemotherapy has improved survival for some types of brain tumors but not for glioma where the prognosis is still poor, while preventing microglial overactivation appears to be an attractive therapeutic approach for various brain tumors. However, therapeutic strategies targeting microglia should consider the dual role of these cells and the produced cytokines and the monitoring of microglial activation during the course of disease might give an indication as to when to initiate anti-tumoral therapies. Taken together, microglial activation and microglia-mediated inflammatory responses play essential roles in the pathogenesis of glioma, elucidation of the complexity, and imbalance of microglial activation may shed light on novel therapeutic approaches for brain tumor immunotherapy.

## 9. Future Perspectives

Clinical studies are needed to learn whether the different immunomodulatory agents are able to achieve adequate therapeutic concentrations in CNS. However, the role of the immunodrugs in glioma progression suggests the great importance to identify new biological compounds to counteract tumor growth. Consequently, considering the properties of immunotherapy could represent alternative treatments for glioma, alone or in association with chemotherapy drugs generally used to improve the quality of life of patients.

## Figures and Tables

**Table 1:** The table summarizes the scientific paper showing the type of tumor that was investigated and the target proteins involved.

Tumor Type	Type of Study	Target/Protein Involved	References
Glioblastoma	Clinical	PD-1	Reardon et al. [101]
Glioblastoma	Clinical	CD4+ and CD8+	Wang et al. [33]
Glioblastoma	Clinical	M1 and NK1	Sagnella et al. [103]
Glioma	Preclinical	IL-2	Glick et al. [110]
Brain	Preclinical	IL-2	Sampath et al. [111]
Glioma	Preclinical	IFN-α	Horton et al. [113]
Glioma	Preclinical	CXCR2/CXCL2	Acker et al. [119]
Glioma	Preclinical	HGF/SF	Badie et al. [58]
Glioblastoma	Preclinical	MerTK	Su et al. [121]
Glioblastoma	Preclinical	EGFR/ CSF-1R	Coniglio et al. [122]
Glioblastoma	Preclinical	TRAIL	Chiu et al. [123]
Glioblastoma	Preclinical	TNF-α	Meisen et al. [124]
